# Supplementary material for: Patterns of Intron Gain and Loss in Fungi
Source: PLoS Biol. 2004 Nov 30;2(12):e422. doi: 10.1371/journal.pbio.0020422 (PMC532390; doi:10.1371/journal.pbio.0020422)
Supplement: Table S1 — Also available at http://genes.mit.edu/NielsenEtAl/. (4.3 MB ZIP). [file pbio.0020422.st001.zip › NielsenEtAl/html/1356.html]

AN0140.1.NCU01756.1.MG03879.1.FG10856.1


```
 CLUSTAL W (1.82) Multiple Sequence Alignments - Introns Inserted


Sequence 1: MG03879.1	422 aa
Sequence 2: FG10856.1	439 aa
Sequence 3: NCU01756.1	430 aa
Sequence 4: AN0140.1	446 aa
Alignment Length: 451 aa
Number Identitical Residues: 353 aa
Alignment Score (without introns) 14609


MG03879.1 	---------MDN2-------G1FAGNDSPSFVFPTAIATKS-AGASGGTGSGRPAVANKP
NCU01756.1	MANTTPAVVMDN2SS--SSAG~FAGNDSPSFVFPTAIATKSPSAGTGGSGSGRPAVANKP
FG10856.1 	MANQTPAVVMDN2GTGFSKLG1FAGNDSPSFVFPTAIATKGPTGGAGGSGSGRPAVANKP
AN0140.1  	MANQTPAVVMDN2GTGYSKLG1FAGNDSPSFVFPTAIATKAGAGSTGG-GS-RPAVANKP
          	 :. :.:  *** .:. :. * ******************. :..:** ** ********

MG03879.1 	SFLTGGAGPGGHLSGKRGTEDLDFFIGDEAIAASSGPG1Y~GLHYPIRHGQIENW0DHME
NCU01756.1	SFLTGGAGPGGHLSAKRGTEDLDYFIGDEAVAAANGPG~T2-------HGQIENW0DHME
FG10856.1 	SFLTGGAGAGGHLSGKRGTEDLDFFIGDEAIAASGGPG1Y~GLHYPIRHGQIENW0DHME
AN0140.1  	SFLS--SGGGGHLSAKRGTEDLDFFIGDEALAAASGPG~Y~GINYPIRHGQIENW~DHME
          	***:  :* *****.********:******:**:.***   .   .  ******* ****

MG03879.1 	RFWSNSIFKYLRVEPEDHFFLLTEP0------------PLNPPENRENTAEIFFESFNCA
NCU01756.1	RFWSNSIFKYLRVEPEDHHFLLTEP0------------PLNPPENRENTAEIFFESFNCA
FG10856.1 	RFWSNSIFKYLRVEPEDHYFLLTEP0------------PLNPPENRENTAEIFFESFNCA
AN0140.1  	RFWSNSIFKYLRVEPEDHYFLLTEP0CLNNHRLITRQQPMNPPENRESTAEIMFESFNCA
          	******************.****** . ..    : ..*:*******.****:*******

MG03879.1 	GLYIAVQAVLALAASWTSSKVHDRSLTGTVIDSGDGVTHVIPVAEGYVIGSSIKSIPIAG
NCU01756.1	GLYIAVQAVLALAASWTSSKVQDRSLTGTVIDSGDGVTHVIPVAEGYVIGSSIKSIPIAG
FG10856.1 	GMYIAVQAVLALAASWTSSKVQDRSLTGTVIDSGDGVTHVIPVAEGYVIGSSIKSIPIAG
AN0140.1  	GLYIAVQAVLALAASWTSSKVTDRSLTGTVIDSGDGVTHVIPVAEGYVIGSSIKSIPIAG
          	*:******************* **************************************

MG03879.1 	RDITYFVQSLLRDRGEPDSSLKTAQEIKEEYCYVCPDIVKEFTKFDRDRSRFARHVVSQP
NCU01756.1	RDITYFVQSLLRDRGEPDSSLKTAQEIKEEYCYVCPDIVKEFAKYDRDRSRFLKHTITQP
FG10856.1 	RDITYFVQSLLRDRGEPDSSLKIAQEIKEEYCYVCPDIVKEFSKYDQDRTRFAKHVVSHP
AN0140.1  	RDITYFVQSLLRDRGEPDSSLKTAERVKEEYCYVSPDIVKEFARYDREPDRFLKHTVTSP
          	********************** *:.:*******.*******:::*::  ** :*.:: *

MG03879.1 	GGREVGVDVGYERFLAPEIFFNPEIYSSDFLTPLPVVVDGVIQSSPIDVRRGLYKNIVLS
NCU01756.1	GGRQVTVDVGYERFMAPEIFFNPEIYSSDFLTPLPVVVDGVIQSSPIDVRRGLYKNIVLS
FG10856.1 	NGRQVSVDVGYERFLAPEIFFNPEIYSSDFLTPLPVVVDGVIQQSPIDVRRGLYKNIVLS
AN0140.1  	NGRSVTIDVGYERFLAPEIFFNPEIYSSDFLTPLPVVVDGVIQSSPIDVRRGLYKNIVLS
          	.**.* :*******:****************************.****************

MG03879.1 	GGSTLYKDFGRRLQRDIKQLVDARIRASEMRSGGAKSGGLEVQVITHKRQRHGPWFGGSL
NCU01756.1	GGSTLYKDFGRRLQRDIKQLVDTRIKASEVRSGGAKSGGLDVQVITHKRQRHGPWFGGSL
FG10856.1 	GGSTLYKDFGRRLQRDIKQLVDARIRASEVRSGGARSGGLDVSVITHKRQRHGPWFGGSL
AN0140.1  	GGSTLYKDFGRRLQRDIRHLVDARIRASEARSGGARSGGLDVAVVTHKRQRHGPWFGGSL
          	*****************::***:**:*** *****:****:* *:***************

MG03879.1 	LGQTPEFRSYCHTKAE0YQEYGPGIVRRFALLGGPGGS
NCU01756.1	LGQTPEFRSYCHTKAE0YQEYGPSIVRRFALLGGPGGS
FG10856.1 	LGQTPEFRSYCHTKAE0YQEYGPSIVRRFALLGGPGGS
AN0140.1  	LGQTPEFRSYCHTKAE~YDEIGPSIVRRFALLGGPGT-
          	**************** *:* **.************
```
